# Chlorophyll Catabolites in Senescent Leaves of the Lime Tree (*Tilia cordata*)

**DOI:** 10.1002/cbdv.201200203

**Published:** 2012-11-19

**Authors:** Mathias Scherl, Thomas Müller, Bernhard Kräutler

**Affiliations:** Institute of Organic Chemistry and Center of Molecular Biosciences, University of InnsbruckInnrain 80/82, A-6020 Innsbruck

**Keywords:** Chlorophyll, Senescence, Pigments, Porphyrins

## Abstract

In cold extracts of senescent leaves of the Lime tree (*Tilia cordata*), two colorless nonfluorescent chlorophyll catabolites (NCCs) were identified, named *Tc*-NCC-1 and *Tc*-NCC-2, as well as a polar yellow chlorophyll catabolite (YCC), named *Tc*-YCC. The constitution of the two NCCs was determined by spectroscopic means. In addition, a tentative structure was derived for *Tc*-YCC. The three chlorophyll degradation products exhibited tetrapyrrolic structures, as are typical of NCCs or YCCs, and turned out to be rather polar, due to a glucopyranosyl group at their 8^2^-position. At their 3-positions, the more polar *Tc*-NCC-1 carried a 1,2-dihydroxyethyl group and the less polar *Tc*-NCC-2 a vinyl group. *Tc*-YCC was identified as the product of an oxidation of *Tc*-NCC-1.

## Introduction

Chlorophyll biosynthesis and chlorophyll breakdown are fascinating natural life processes on earth 1, which can be observed from outer space 2. Indeed, an estimated 10^9^ tons of chlorophyll are formed and degraded every year on earth 3 4. Strikingly, chlorophyll breakdown and the appearance of autumnal fall colors have remained a stunning mystery 4–6 until about twenty years ago, when a first colorless tetrapyrrolic chlorophyll catabolite 1a *(Hv-NCC-1)* was identified in senescent primary leaves of barley 7 8. Nonfluorescent chlorophyll catabolites (NCCs) were subsequently also found in other plants, and they were assumed to represent the final stages of chlorophyll catabolism in senescent leaves 2 6 9 10. Since then, over a dozen NCCs were detected in higher plants, and their structures were analyzed *(Scheme* and Table 1).

**Table:** *Structures of Nonfluorescent Chlorophyll Catabolites Found in Senescent Leaves of Higher Plants* (the labels R^1^ to R^4^ refer to the general constitutional formula of NCCs in the *Scheme*; atoms numbered according to their original position in chlorophyll a)

No.^a^)	R^1^^b^)	R^2^	R^3^	R^4^	C(1)^c^)	Provisional names^d^)	Ref.
1a	OH	Me	HOCH_2_CH(OH)	Me	n	*Hv*-NCC-1	[7][8]
1b	OH	Me	HOCH_2_CH(OH)	Me	epi	*So*-NCC-2	[11] [12]
2	H	Me	CH_2_ = CH	Me	epi	*Cj*-NCC-2/*So*-NCC-5	[12][13]
3a	OH	Me	CH_2_ = CH	Me	n	*Sw*-NCC-58	[14]
3b	OH	Me	CH_2_ = CH	Me	epi	*Cj*-NCC-1/*So*-NCC-4/*Pc*-NCC-2/*Md*-NCC-2	[12][13][15][16]
4a	b-GlcO	Me	CH_2_ = CH	Me	n	*At*-NCC-4	[17]
4b	b-GlcO	Me	CH_2_ = CH	Me	epi	*Nr*-NCC-2/*Zm*-NCC-2/*Pc*-NCC-1/*Md*-NCC-1/*Tc*-NCC-2	[16][18][19]^e^)
5	b-GlcO	Me	HOCH_2_CH(OH)	Me	epi	*Zm*-NCC-1/*Tc*-NCC-1	[19]^e^)
6	b-(6’-O-Mal) GlcO	Me	CH_2_ = CH	Me	epi	*Nr*-NCC-1	[18]
7	H	H	CH_2_ = CH	Me	n	*Bn*-NCC-4/*At*-NCC-5	[17]
8	H	H	CH_2_ = CH	HOCH_2_	n	*At*-NCC-3	[17][20]
9a	OH	H	CH_2_ = CH	Me	n	*Bn*-NCC-3/*At*-NCC-2	[17][21]
9b	OH	H	CH_2_ = CH	Me	epi	*So*-NCC-3	[12]
10	OH	H	HOCH_2_CH(OH)	Me	epi	*So*-NCC-1	[12]
11	MalO	H	CH_2_ = CH	Me	n	*Bn*-NCC-1	[21] [22]
12	b-GlcO	H	CH_2_ = CH	Me	n	*Bn*-NCC-2/*At*-NCC-1	[17] [21]

a )Compound number.

b )Abbreviations: Mal = malonyl; Glc = glucopyranosyl.

c ) Configuration at C(1) from correlation with NCCs derived from *p*FCC (n = ‘normal’) or from *epi-p*FCC (epi = ‘epimeric’), the absolute configuration at C(1) is still unknown.

d ) *Hv*-NCC-1 (1a; from barley, *Hordeum vulgare* [7] [8]), *So*-NCCs (1b, 2, 3b, 9b, and 10; from spinach, *Spinacia oleracea* [11] [12]), *Cj*-NCCs (2 and 3b; from Katsura tree, *Cercidiphyllum japonicum* [13] [15]), *Sw*-NCC-58 (3a; from Peace Lily, *Spathiphyllum wallisii* [14]), *Pc*-NCCs (3b and 4b; from *Pyrus communis* [16]), *Md*-NCCs (3b and 4b; from *Malus domestica* [16]), *At*-NCCs (4a, 7, 8, 9a, and 12; from *Arabidopsis thaliana* [17] [20]), *Nr*-NCCs (4b and 6; from tobacco, *Nicotiana rustica* [18]), *Zm*- NCCs (4b and 5; from maize, *Zea mays* [19]), and *Bn*-NCCs (7, 9a, 11, and 12; from oilseed rape, *Brassica napus* [21] [22]), and *Tc*-NCCs (5 and 4b; from Lime tree (*Tilia cordata*), this work).

e ) This work.

About ten years ago, two ‘urobilinogenoidic’ chlorophyll catabolites were discovered in extracts of senescent primary leaves of barley 23, *i.e.,* linear tetrapyrroles, which were considered to represent putative products of further breakdown of *Hv-NCC-1* (1a) by an oxidative deformylation at ring *B.* Evidence for another type of further oxidative transformation of NCCs was also provided more recently by the yellow chlorophyll catabolites (YCCs) and pink chlorophyll catabolites (PiCCs) detected in senescent leaves of the Katsura tree *(Cercidiphyllum japonicum)*, which were identified as dehydrogenation products of the tetrapyrrolic NCC 3b *(Cj-*NCC-1) 24 25. All of these findings were consistent with an essentially ‘linear’ path of chlorophyll breakdown in higher plants (see the Scheme) 26.

**Scheme:**
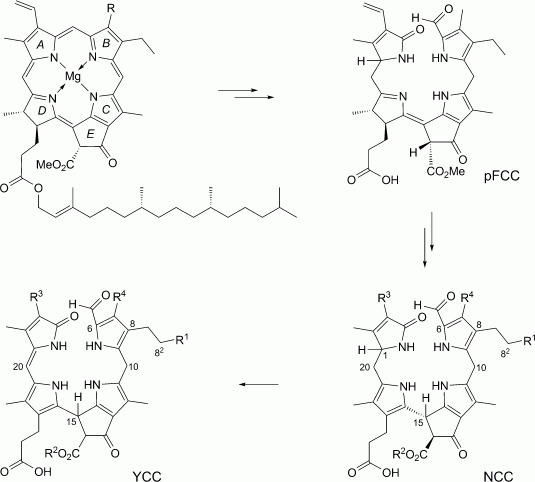
*Outline of the Main Path of Chlorophyll Breakdown in Higher Plants, Including Structural Formulae of Chlorophylls a and b, of the Primary Fluorescent Chlorophyll Catabolite* (*p*FCC), *of Non-fluorescent Chlorophyll Catabolites* (NCCs), *and of Yellow Chlorophyll Catabolites* (YCCs; with general formulae for NCCs and YCCs, see *Table 1* for specific constitutional formulae of individual NCCs). Atom numbering used for chlorophylls according to *IUPAC* (see, *e.g.,* 1)

However, several recent observations suggested the existence of divergent pathways of chlorophyll breakdown in higher plants. A strikingly contrasting stereo-chemical variant of the ‘urogenobilinoidic’ catabolites from barley was found in naturally de-greened leaves of Norway Maple *(Acer platanoides),* classified as dioxobilanes, and indicating a different path to these tetrapyrroles 27. Structurally divergent, ‘hypermodified’ blue fluorescent chlorophyll catabolites (FCCs) were observed as remarkably persistent breakdown products in banana *(Musa acuminata)* fruits 28 and leaves 29, as well as in senescent leaves of the ‘peace Lily’ *(Spathiphyllum wallisii),* a tropical evergreen 14.

Here, we describe an investigation of chlorophyll breakdown products in senescent leaves of the lime tree *(Tilia cordata),* a first representative of the genus *Tilia* (Malvaceae) to be investigated in this respect. Lime trees are well-known deciduous trees native to the forests of the northern hemisphere in Europe, Asia, and Eastern North America and Central America 30. A reference to the importance of *Tilia* sp. in central Europe can be found in the Middle High German ‘*Nibelungenlied',* where a lime tree leaf ('linden leaf) covered *Siegfried's* back during his bath in the blood of a wounded dragon and gave rise to his vulnerable spot^1^). Beside a special medicinal relevance of lime trees (colds, cough, fever, *etc.* are often treated with extracts of this plant 31), representatives of these deciduous trees became increasingly important in municipal parks of cities and play a central role as avenue trees 30.

## Results and Discussion

**Fig 1:**
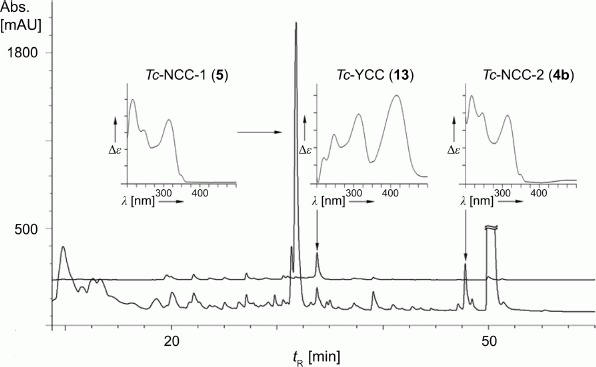
*HPLC Analysis of an extract of senescent leaves of* Tilia cordata (lower trace: detection at 320 nm, trace above: detection at 420 nm). For details, see the *Exper. Part. Insets:* Online UV/VIS spectra of *Tc-NCC-1* (5; left), *Tc-YCC* (13; middle), and Tc-NCC-2 (4b; right).

**Fig 2:**
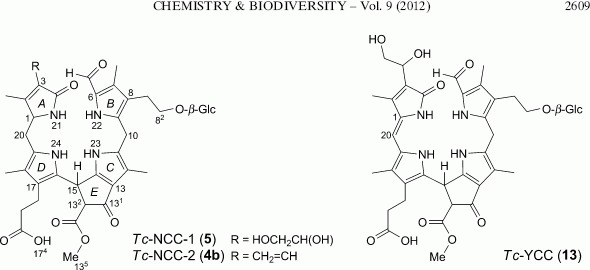
*Constitutional formula of* Tc-*NCC-1* (5), Tc-YCC (13), *and* Tc-*NCC-2* (4b). Numbering of heavy atoms according to their original atom numbering in chlorophylls.

In a cold MeOH extract of yellow (senescent) fall leaves of a lime tree *(Tilia cor data),* two polar colorless and nonfluorescent chlorophyll catabolites (NCCs) and a yellow chlorophyll catabolite (YCC) were provisionally identified by analytical HPLC, on the basis of their characteristic UV-absorbance properties (see Fig. 1 24 32. To inhibit adventitious oxidation of NCCs to yellow catabolites such as Tc-YCC (13; Fig. 2) 24 during isolation and workup, the freshly picked senescent leaves were immediately frozen with liquid N_2_ and directly analyzed by HPLC. The different HPLC retention times on the stationary ‘reversed’ phase (f_R_(Tc-NCC-1 (5)) 31.8 min, *t_R_ (Tc-YCC* (13)) 33.8 min, and *t_R_* (Tc-NCC-2 (4b)) 47.8 min) reflected the different polarities of the three catabolites.

UV/VIS and CD spectra of the NCCs **4b** and **5** matched those of the chlorophyll catabolite *Hv-NCC-1* (**1a**). An absorbance maximum near 314 nm indicated the presence of an α-formyl-pyrrole moiety at ring *B* 7 32. The yellow tetrapyrrolic catabolite Tc-YCC (13) exhibited a characteristic additional maximum at 415 nm, consistent with its color and suggesting (Z)-configuration for its C(20)=C(l) bond 24 25 (see Fig. 1).

For further structural analysis, 100 g (wet weight) of senescent lime tree leaves were extracted according to a three-stage purification procedure based on a cold extraction, followed by separation by MPLC and by preparative HPLC (for details, see the *Exper. Part)* to give 5.1 mg (6.1 mmol) of Tc-NCC-1 (**5**), 1 mg (1.2 mmol) of the less polar *Tc-NCC-2* (**4b**), and 0.2 mg of the yellow catabolite Tc-YCC (13) (determined by UV/VIS spectroscopy).

**Fig 3:**
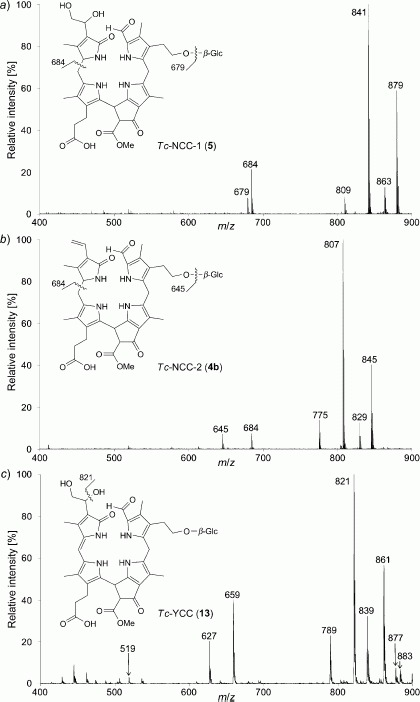
*Positive-ion-mode LC/ESI-MS of a) Tc-NCC-1* (5), b) *Tc-NCC-2* (4b), *and* c) *Tc*-YCC (13) *with corresponding constitutional formulae*

Mass spectrometry was utilized to derive a tentative molecular formula of Tc-NCC-1 (5) as C_41_H_52_N_4_O_15_ (see Fig. 3, a). ESI-MS in the positive-ion mode showed a signal at *m/z* 841.49 which corresponded to the pseudo-molecular ion C_41_H_53_N_4_O_15_^+^ *([M*+H]^+^; calc. 841.35) of Tc-NCC-1 (**5**). In the mass spectrum, characteristic fragment-ion peaks at *m/z* 809.5, 684.4, and 679.3 were also detected, which corresponded to loss (from *[M*+H] ^+^ ) of MeOH, as is typical of the methyl ester functionality, to loss of ring *A,* and to loss of the sugar moiety (as [C_6_H_10_O_5_]), respectively.

A 500-MHz ^1^H-NMR spectrum of a solution of Tc-NCC-1 (5) in CD_3_OH revealed signals of 44 of the 52 H-atoms: a *singlet* (at low field) of the formyl H-atom, four *singlets* (at high field) of the four Me groups attached at the b-pyrrole positions, and a *singlet* at 3.74 ppm (due to a methyl ester function). In addition, the signals of the H-atoms H-N(21), H-N(24), and H-N(23) were detected between 8.0 and 11.3 ppm, and could be assigned with the help of COSY, ROESY, and HMBC data. However, in contrast to the ^1^H-NMR spectrum of *Tc*-NCC-2 (**4b**), the typical signals for a peripheral vinyl group in the intermediate field range were absent. ^1^H,^13^C-Hetero-nuclear NMR correlations (from HSQC and HMBC spectra 33 of *Tc*-NCC-1 (5) in CD_3_OH) allowed assignment of the signals of a 1,2-dihydroxyethyl side chain. ^1^H,^1^H-Homonuclear correlations from ROESY spectra and 1H,13C-heteronuclear correlations from HMBC spectra 33 indicated C(8^2^) as the site of the attachment of the sugar moiety. The shifts of the ^1^H and ^13^C signals for the CH_2_(8^2^) group were consistent with an O-bridge to the peripheral sugar substituent. The latter was identified as a b-glucopyranoside unit by comparing the ^1^H and ^13^C chemical shifts with the spectra of methyl b-d-glucopyranoside 34, as well as by comparing the NMR spectra of *Tc-*NCC-1 (5) with those of Bn-NCC-2, Nr-NCC-2, and Zm-NCC-1, where a peripheral *b-*glucopyranosyl group at C(8^2^) had also been found 18 19 22. The signal of H-C(15) appeared as a *doublet* (J(H,H) = 2.5 Hz, in CD_3_OH) due to coupling with H-C(13^2^). Therefore, a relative trans-configuration of H-C(13^2^) and H-C(15), which is typical for stable isomers of NCCs 13 32, in *Tc*-NCC-1 was derived using the *Karplus* relation 34.

ESI-MS in the positive-ion mode was also used to deduce the tentative molecular formula of *Tc*-NCC-2 (4b) as C_41_H_50_N_4_O_13_ (see Fig. 3, b). The peak at *m/z* 807.37 corresponded to the pseudo-molecular ion C_41_H_51_N_4_O+13 *([M*+H]^+^; calc. 807.34). The basic substitution pattern of the tetrapyrrolic core of *Tc*-NCC-2 could again be determined by the analysis of characteristic fragment-ion peaks at *m/z* 775.3, 684.3, and 645.3 due to loss of MeOH, of ring *A,* and of the sugar moiety (as [C_6_H_10_O_5_]), respectively.

**Fig 4:**
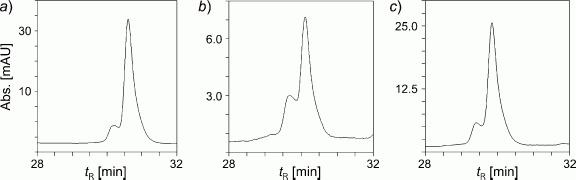
*HPLC Analyses of a) *Tc*-NCC-1,* b) *ofZm-NCC-1, and* c) ofa 1:2 *mixture of the two NCCs.* In the samples of *Tc*-NCC-1 and Zm-NCC-1, a second fraction *(ca.* 10%) occurred in addition to the main fraction (presumably, the minor fraction is the *13*^2^-epimer of the main NCCs, due to epimerization at C(13^2^) of the β-keto ester functionality).

A 600-MHz ^1^H-NMR-spectrum of 4b (in CD_3_OD) exhibited signals for 40 of the 50 H-atoms: a *singlet* (at low field) for the formyl H-atom, four *singlets* (at high field) for the four Me groups in the b-pyrrole positions, and a *singlet* at 3.74 ppm (due to the methyl ester function). In addition, the typical signal pattern for a vinyl group was detected around 6 ppm. 1H,^1^3C-Heteronuclear correlations (HSQC and HMBC 33) allowed the assignment of all ^1^H and ^13^C signals (see *Exper. Part).* A sugar moiety was identified as a b-glucopyranosyl group at C(8^2^) from ^1^H,^1^H-ROESY correlations, as well as 1H,^1^3C-heternuclear correlations from HMBC spectra 33. Again, the indicated site of attachment for the peripheral glucopyranosyl substituent (C(8^2^)) *via* a bridging O-atom, O(8^3^), was consistent with the typical downfield shifts of the ^1^H and ^13^C signals of CH_2_(8^2^). The lack of the signal for H-C(13^2^) in the ^1^H-NMR spectra (in CD_3_OD) of *Tc*-NCC-2 (4b) indicated the exchange-labile a-position of the b-keto ester functionality at ring *E* to have undergone H/D exchange. HPLC Co-injection of *Tc*-NCC-1 (5) and of the constitutionally identical analog from maize, Zm-NCC-1 19, indicated a common t_R_ of *ca.* 30 min, suggesting the two NCCs to be identical (see Fig. 4 in the *Exper. Part;* for details of co-injection experiments, see the *Exper. Part).*

The CD spectra of the *Tc*-NCCs **5** and **4b** were consistent with the suggested common configuration of C(15) in naturally occurring NCCs from higher plants 7 26 32. The stereogenic center C(15) has been proposed to result from a nonenzymatic, stereoselective isomerization of fluorescent chlorophyll catabolites 35 36 to the corresponding NCCs 13. The relative configuration at C(15) and C(13^2^), with H-C(15) *cis* to the COOMe group at C(13^2^) in (the prevailing epimer of) **5** and **4b** is a likely result of a nonenzymatic equilibration reaction at the acidic b-keto ester position C(13^2^), which adjusts its configuration to that at C(15). The latter process would occur in the vacuoles of senescent plant cells 13 37, but it also takes place when isolated NCCs are kept in a protic solution.

A tentative molecular formula of *Tc*-YCC (**13**) could be deduced as C_41_H_50_N_4_O_15_ by LC/ESI-MS in the positive-ion mode (see Fig. 3, c), which showed a prominent peak at *m/z* 839.1 of the pseudo-molecular ion C_41_H_51_N_4_O_15_+ (*[M+*H] +; calc. 839.33). The mass spectrum of **13** showed characteristic fragment-ion peaks at *m/z* 821.1,789.3, and 659.2, due to loss of H_2_O (from the 1,2-dihydroxyethyl group at ring *A),* loss of MeOH, and combined loss of the sugar moiety and H_2_O (in total [C_6_H_12_O_6_]), respectively. The loss of ring *A,* a typical fragmentation pathway of NCCs (see *Fig. 3),* could not be observed in the mass spectrum of *Tc*-YCC (13), compatible with the C=C bond at ring *A.* For further characterization of 13 as oxidation product of *Tc*-NCC-1 (**5**), a nearly colorless spot of analytically pure **5** on a silica-gel TLC plate was irradiated for 10 min with a 365-nm UV lamp 25, whereupon the ‘spot’ acquired a brownish-red color (see *Exper. Part).* A major yellow product fraction formed, which had a UV/VIS spectrum typical for a YCC with (Z)-configuration of the C(l)=C(20) bond 24 25, and which was shown by analytical HPLC to co-elute with the yellow catabolite *Tc*-YCC **(13).**

## Conclusions

In the present work, non-green chlorophyll catabolites were analyzed in fresh extracts of naturally de-greened lime tree (*Tilia cor data)* leaves, a first representative of the genus *Tilia* to be investigated in this respect. Two colorless nonfluorescent chlorophyll catabolites (NCCs) were identified in the senescent leaves, *Tc*-NCC-1 (**5**) and *Tc*-NCC-2 (**4b**), and their structures were characterized. The basic build-up of **4b** and **5** was found to be the same as known for NCCs from higher plants. The available NMR data revealed the functionalization at C(8^2^) of the *Tc*-NCCs to imply a common β-glucopyranosyl moiety, as in Bn-NCC-2 (12) from oilseed rape *(Brassica napus),* in _N_r-NCC-2 (**4b**) from tobacco, and in Zm-NCCs **4b** and **5** from senescent maize leaves *(Zea mays).* Furthermore, the spectroscopic data indicated the tetrapyrrolic cores of the two *Tc*-NCCs **5** and **4b** to have a common relative configuration at the stereogenic centers C(1), C(13^2^), and C(15). The absolute configuration at C(1) was deduced to be the same in the *Tc*-NCCs **5** and **4b** as in the glucosylated _N_r-NCCs **4b** and 6, and Zm-NCCs **4b** and 5. Therefore, the absolute configuration at C(1) was indicated to be opposite to that in Bn-NCC-2 (12). NCCs (and FCCs) fall into two classes with respect to the configuration at C(1) due to the evolution of two types of RCC-reductases (RCCR) in higher plants 36 38–40. Hence, it was of interest to note the structural relationships with respect to the type of peripheral functionalization for the NCCs and of their configuration at C(1). The indicated availability of two closely related NCCs, *Tc*-NCC-1 and *Tc*-NCC-2, in senescent lime tree leaves parallels the occurrence of NCCs in other senescent plants, where a range of peripheral groups was observed 32 41. Our studies on the *Tc*-NCCs provide further support for the view that the main constitutional variations of the chlorophyll catabolites in various higher plants involve enzyme catalyzed, peripheral refunctionalization reactions. The peripheral adaptions with polar functions have been suggested to be of relevance for the transport and for the final deposition of chlorophyll catabolites in the vacuoles 6 10 37 42.

A yellow chlorophyll catabolite *Tc*-YCC (13) was identified in fresh extracts of lime tree leaves, and it was suggested to be a product of a naturally occurring oxidation reaction of **5** during senescence. Our study, therefore, provides (further) evidence for the notion that NCCs may not generally be the ‘final products’ of chlorophyll in senescent plants 41. All these findings strengthen the view that, while the pathways of chlorophyll catabolism in various higher plants may be closely related, they follow divergent branches, when including functional details. Indeed, the natural formation of the colored and photoactive YCCs such as of 13 would not be consistent with the role of chlorophyll breakdown as a mere ‘detoxification’ process either.

We would like to thank *C. Kreutz* for acquiring the NMR-spectra, and *Hans-Jçrg* and *Hildegard Patscheider* for giving us access to the *Hotel Linde* lime tree. Financial support by the *Bundesministerium fur Wissenschaft und Forschung* (BM.W_F, Project SPA/02-88/Recycling the Green to *T. M.*) and by the *Austrian Science Foundation (FWF,* Project No. L-472 to *B. K.*) is gratefully acknowledged.

## Experimental Part

*General.* Commercial solvents (reagent-grade) were redistilled before use for extractions. HPLC-Grade MeOH and Et_2_O were purchased from *Merck* (DE-Darmstadt) and *Acros Organics* (B-Geel). Potassium dihydrogen phosphate,*puriss. p.a.,* potassium phosphate dibasic-anhydrous,*puriss. p.a.,* and ammonium acetate,*puriss. p.a.,* were from *Fluka* (CH-Buchs).

Five-g and 1-g *Sep-Pak-C18* cartridges were from *Waters Associates.* The pH values were measured with a *WTW Sentix 21* electrode connected to a *WTW pH_53_5* digital pH-meter.

*HPLC: Dionex Summit* HPLC system with manual sampler, *P680* pump, online degasser and diode array detector, 1-ml or 20-μl injection loop. Data were collected and processed with Chromeleon V6.50.

a) Anal. HPLC: *Phenomenex HyperClone ODS* **5** μm 250 x 4.6-mm i.d. column at 20° protected with a *Phenomenex ODS* 4 mm x 3-mm i.d. pre-column was used with a flow rate of 0.5 ml min^−1^. Solvent *A:*50 mm aq. potassium phosphate (pH 7.0), solvent *B:* MeOH; sovent composition *(A/B)* as function of time (0-75 min): 0-5,80:20; 5-55,80:20 to 30:70; 55-60,30:70 to 0:100; 60-70,0:100;70-75,0:100 to 80:20.

b) Prep. HPLC: *Phenomenex HyperClone ODS* **5** μm 250 x 21.2-mm i.d. column at 20° protected with a *Phenomenex ODS* 10 mm x 5-mm i.d. pre-column was used with a flow rate of **5** and 7 ml min^−1^. Solvent *A:* 50 mm aq. potassium phosphate (pH 7.0), solvent *B:* MeOH.

*MPLC: Buchi* MPLC system equipped with two *C-605* pumps, a *C-615* pump manager, and a *C-630* UV-detector at 254 nm. A 460 x 55-mm i.d. column at 208 filled with *Phenomenex Spepra-C18-E,* 50 mm, 65A was used with a flow rate of 10 ml min^−1^. Solvent *A:* 50 mm aq. potassium phosphate (pH7.0), solvent *B:* MeOH.

*LC/MS: a)* HPLC: *Dionex Ultimate* HPLC system with manual sampler, He degasser, and diode array detector; 20 μl injection loop; *Phenomenex HyperClone ODS* **5** mm 250 x 4.6-mm i.d. column at 20° protected with a *Phenomenex ODS* 4 mm x 3-mm i.d. pre-column was used with a flow rate of 0.5 ml min^−1^. Solvent *A:* 10 mm ammonium acetate (pH 7.0), solvent *B:* MeOH; solvent composition *(A/B)* as function of time (in 75 min): 0-5, 80:20; 5-55, 80:20 to 30:70; 55-60, 30:70 to 0:100; 60-70, 0:100; 70-75, 0:100 to 80:20. Data were collected and processed with Chromeleon V6.50.

*b)* Mass Spectrometry: *Finnigan MAT 95,* electrospray ionization (ESI) source, positive-ion mode, 1.4 kV spray voltage. MS and MS/MS: *Finnigan LCQ Classic,* ESI source, positive-ion mode, 4.5-kV spray voltage (rel. abundance).

*Spectroscopy.* UV/VIS Spectra: *Hitachi U-3000* spectrophotometer; λ_max_ [nm] (log e/rel. *E).* CD Spectra: *JASCO J715;* λ_max_ and *λ* [nm], *De. ^1^H-* and ^13^C-NMR: *Varian Unity Inova 500MHz* spectrometer; *Bruker 600 MHz Avance II+* (d(C1HD2OH) 3.31 ppm, and d(^13^CD_3_OD) 49.0 ppm) 43; <5 in ppm, δ in Hz.

*Analysis of Chlorophyll Catabolites in Senescent Leaves by Anal. HPLC.* Freshly picked lime tree leaves were collected from a lime tree grown near the Hotel Linde (Innsbruck; see illustration for the *Table of Contents)* and immediately stored in liquid N_2_. A leaf (with the area of *ca.* 20 cm^2^) was grounded in a mortar and extracted with 2 ml of MeOH. The resulting suspension was centrifuged **5** min at 13,000 rpm. The methanolic supernatant was diluted with 50 mm aq. potassium phosphate (pH 7.0) 80:20 (v/v). After centrifugation for **5** min at 13,000 rpm, 1 ml of the extract was injected into the anal. HPLC system, to be analyzed with parallel detection at 320 and 420 nm (see Fig. 1).

*Determination of Chlorophyll in Green and Senescent* (yellow) *Lime Tree Leaves by UV/VIS Spectroscopy, of Nonfluorescent and of Yellow Chlorophyll Catabolites* (NCCs and YCC, resp.) *in Senescent* (yellow) *Leaves by Anal. HPLC. Chlorophyll a and b in Green Leaves. A* total area of 9 cm^2^ was cut out of a green lime tree leaf. The leaf was frozen in liquid N_2_, pulverized in a mortar, and extracted with MeOH. The slurry was filtered through a sintered glass filter, and the residue was grounded in a mortar and extracted with MeOH. The procedure was repeated, until the residue was colorless. The MeOH extracts were combined and diluted with MeOH to 100.00 ml in a volumetric flask. The extracts were analyzed by UV/VIS spectrometry. In green *Tilia cordata* leaves, 72.09 ± 3.93 mg·cm”^2^ (80.37 ± 4.38 nmol · cm^−2^) of chlorophyll a and b were found *(n =* 4), the data analysis was based on 44.

*Chlorophyll a and b in Senescent* (Yellow) *Leaves. A* total area of 99 cm^2^ was cut out of eleven senescent lime tree leaves. The extraction and the UV/VIS analysis were performed as described above. Yellow *Tilia cordata* leaves were found to contain 0.55±0.10 μig·cm”^2^ (0.61 ±0.11 nmol·cm^−2^) of residual chlorophyll a and b.

*NCCs in Senescent* (Yellow) *Leaves.* The quantification of the *Tc*-NCCs was accomplished by anal. HPLC. An anal. pure sample of Cj-NCC-1 from *Cercidiphyllum japonicum* was used to prepare a standard soln. (for isolation, see 24; *ε^320^ =* 17,000 as described in 15). Senescent *Tilia cordata* leaves were found to contain 42.2±4.2 μg cm”^2^ (50.2±5.0 nmol-cm-^2^) of *Tc*-NCC-1, 3.7±1.1 u,g-cm-^2^ (4.6± 1.4nmol-cm-^2^) of *Tc*-NCC-2, and 1.6±0.2 ug-cm-^2^ (1.9±0.3 nmol-cm-^2^) of *Tc-YCC* (YCC-content was calculated using e^310^ = 22,400, see 24). These values indicate, in a green lime tree leaf, a total conversion of chlorophyll a and b to NCCs and YCC of >70.4%.

*Collection, Isolation and Structure Elucidation of *Tc*-NCCs.* Senescent *Tilia cordata* leaves were collected at the main campus of the University of Innsbruck in October 2009 and stored at —80°. Senescent (yellow) leaves (100 g (wet weight)) were frozen in liquid N_2_, crushed into small pieces, and freeze-dried *in vacuo* for 3 d, resulting in a dry-weight of *ca.* 40 g. The dried leaves were pulverized using liquid N_2_ and a 300-W blender, and extracted with 70 ml of MeOH. The suspension was filtered with suction over a *Buchner* funnel. The extraction was repeated using another 70 ml of MeOH. The combined 140 ml of MeOH extracts were added portionwise to 350 ml of Et_2_O to precipitate a raw product that was enriched in chlorophyll catabolites. The raw product was cooled in an ice-bath for 15 min. After filtration with suction the white precipitate was dried *in vacuo* (dry weight: 1.77 g) and stored at -80° for further purification by MPLC: The crude product was dissolved in 48 ml of aq. potassium phosphate buffer soln. (50 mm; pH 7) using an ultrasonic bath. After filtration with a *Sartorius* filter two aliquots of the filtrate (24 ml of clear red-brownish solutions) were injected into the MPLC system (flow rate: 10 ml min^−1^); solvent *A:* 50 mm aq. potassium phosphate (pH 7.0); solvent *B:* MeOH; solvent composition *(A/B)* as function of time (0-120 min): 0-90, 70:30; 90-120, 70:30 to 0:100.

The fractions containing *Tc*-NCC-1 and *Tc*-NCC-2 were collected after 50 and 105 min, resp. The collected fractions were concentrated using a rotary evaporator, freeze-dried *in vacuo,* and stored at - 808 for further purification.

The (more-polar) fraction containing *Tc*-NCC-1 (**5**) was dissolved in 0.2 ml MeOH and 2.5 ml of H_2_O using an ultrasonic bath. After filtration of the suspension through a *Sartorius* filter the sample was divided in three aliquots and applied to prep. HPLC; injection vol., 1 ml; flow rate, 7 ml min^−1^; solvent *A:* 50 mm aq. potassium phosphate (pH 7.0); solvent *B:* MeOH; solvent composition *(A/B)* as function of time (0-198 min): 0-180, 90:10 to 65:35; 180-185, 65:35 to 0:100; 185-195, 0:100; 195-198 0:100 to 70:30. Three consecutive prep. HPLC runs were performed, and fractions containing *Tc*-NCC-1 were collected between 62.5 and 68 min. For de-salting, the aq. soln. was then applied to a pre-conditioned *Sepak* cartridge, washed with 20 ml of H_2_O, and eluted with a minimum amount of MeOH. After the sample was dried under high vacuum, 5.1 mg of anal. pure *Tc*-NCC-1 (**5**) were obtained.

The (less-polar) *Tc*-NCC-2 (**4b**) fraction was likewise dissolved in 0.6 ml of MeOH and 2.4 ml of H_2_O using an ultrasonic bath. After centrifugation for 3 min at 13,000 rpm, prep. HPLC (injection vol., 1 ml; flow rate, **5** ml min^−1^) was applied for three aliquots; solvent *A:* 50 mm aq. potassium phosphate (pH 7.0); solvent *B:* MeOH; solvent composition *(A/B)* as function of time (0-180 min): 0-5, 80:20; 5-120,80:20 to 35:65; 120-180: 35:65 to 0:100. Three consecutive prep. HPLC runs were performed, and fractions containing *Tc*-NCC-2 were collected at 79 min. For de-salting, the aq. soln. was then applied to a pre-conditioned *Sepak* cartridge, washed with 20 ml of H_2_O, and eluted with a minimum amount of MeOH. The solvents were removed using a rotary evaporator. To obtain 1.1 mg of an anal. pure *Tc*-NCC-2, one more prep. HPLC run (the sample was dissolved 0.2 ml of MeOH and 0.6 ml of H_2_O), followed by *Sep-Pak* desalting (see above) had to be performed.

*Data ofTc-NCC-1* (**5**). *t_R_* 31.8 min (see main text and *Fig. 1*). UV/VIS (MeOH, c = 7.M0-^s^ M): 245 (4.16), 314 (4.23). CD (MeOH, c = 2.5-10-^s^ M); 226 (18), 258sh (-6), 281 (-17), 315 (3). ^1^H-NMR (500 MHz, CD_3_OH, 108): 1.91 *(s,* Me(^1^81)); 2.04 *(s,* Me(2^1^)); 2.10 *(s,* Me(^1^21)); 2.23 *(s,* Me(7^1^)); 2.25-2.36 (m, CH_2_(17^2^)); 2.44 *(dd,* 7=10.1, 14.3, H_a_-C(20)); 2.57-2.67 (m, *H_a_-C(17^1^),* CH_2__(_8^1^)); 2.68-2.77 (*m*, H_b_-C(17^1^)); 2.90 *(dd,* 7=3.8,14.3, H_b_-C(20)); 3.16 *(t-like,* H-C(2')); 3.23-3.29 (m, H-C(4'), H-C(5')); 3.32-3.40 (m, H_a_-C(8^2^), H-C(3')); 3.60-3.73 (m, CH_2_(3^2^), H_a_-C(6'), H_b_-C(8^2^)); 3.74 *(s,* Me(13^5^)); 3.78 (br. *d,* J = 2.5, H-C(13^2^)); 3.84 (d, 7= 11.8, H_b_-C(6')); 3.94-4.09 (m, H-C(l), CH_2_(10)); 4.16 *(d,* 7=7.8, H-C(l')); 4.56 *(t-like,* H-C(3^1^)); 4.87 (br. d,J = 2.5, H-C(15)); 8.04 *(s,* H-N(21)); 9.30 *(s,* H-C(**5**)); 9.42 *(s,* H-N(24)); 11.25 *(s,* H-N(23)). 1^3^C-NMR (125 MHz, CD_3_OH, 108; ^13^C-signal assignment from HSQC and HMBC experiments): 8.2 (C(7^1^); 9.1 (C(18^1^)); 9.1 (C(12^1^)); 12.3 (C(2^1^)); 21.8 (C(17^1^)); 23.7 (C(10)); 24.9 (C(81)); 29.4 (C(20)); 37.1 (C(15)); 39.3 (C(17^2^)); 52.6 (C(13^5^)); 62.4 (C(6')); 62.6 (C(1)); 65.9 (C(3^2^)); 67.9 (C(13^2^)) 68.4 (C(3^1^)); 70.3 (C(8^2^));71.2 (C(4')); 75.1 (C(2')); 77.5 (C(5'));77.9 (C(3')); 104.0 (C(1')); 112.3 (C(12)); 115.0 (C(18)); 120.4 (C(17)); 120.5 (C(8)); 124.4 (C(16)); 124.4 (C(19)); 125.9 (C(13)); 129.0 (C(6)); 131.4 (C(3)); 134.2 (C(11)); 135.3 (C(7)); 139.9 (C(9)); 158.7 (C(2)); 161.5 (C(14)); 171.4 (C(13^3^)); 175.1 (C(4)); 176.8 (C(**5**)); 181.4 (C(17^3^)); 191.6 (C(13^1^)). ESI-MS: 879.43 (60, ), 863.49 (17, [M + Na] + ), 841.49 (100, [M+H] + ), 809.47 (11, [M + H-MeOH] + ), 684.35 (22, ringA] + ), 679.33 (11, [M + H-QH_10_O_s_] + ).

*Data of*Tc*-NCC-2* (**4b**). *t_R_* 47.8 min (see main text and *Fig.1).* UV/VIS (50 mm aq. potassium phosphate (pH7.0)/MeOH 65:35, c = 2.6-10-^s^ M): 240 (1.0), 312 (0.83). CD (50 mm aq. potassium phosphate (pH7.0)/MeOH 65:35, c = 2.6-10-^s^ M): 225 (26), 262sh (9), 281 (-19), 318 (3). ^1^H-NMR (600 MHz, CD3OD, 108): 1.92 *(s,* Me(18^1^)); 1.99 *(s,* Me(2^1^)); 2.14 *(s,* Me(12^1^)); 2.21 *(s,* Me(7^1^)); 2.29-2.38 (m, H_a_-C(20), CH_2_(17^2^)); 2.54-2.68 (m, CH_2__(_8^1^), H_a_-C(17^1^)); 2.74–2.81 (m, H_b_-C(17^1^)); 2.89 *(dd,* 7= 4.2,14.2, H_b_-C(20)); 3.17 *(t-like,* H-C(2')); 3.24-3.28 (m, H-C(4'), H-C(5')); 3.33-3.36 (m, H_a_-C(8^2^)); 3.42 *(t-like,* H-C(3')); 3.64 *(dd,* 7=4.9, 12.0, H_a_-C(6')) superimposed by 3.67 *(m,* H_b_-C(8^2^)); 3.74 *(s,* Me(13^5^)); 3.84 (d,7=12.0, H_b_-C(6')); 3.92-4.13 *(m,* H-C(l), CH_2_(10)); 4.16 (rf,7=7.8, H-C(l')); 4.88 (br. *s,* H-C(15)); 5.35 (d-like,J = 11.7, H_a_-C(3^2^)); 6.12 (d-like,7= 17.8, H_b_-C(3^2^)); 6.46 *(dd,*7= 11.7,17.8, H-Cp^1^)); 9.27 *(s,* H-C(**5**)). 1^3^C-NMR (125 MHz, CD_3_OD, 108; ^13^C-signal assignment from HSQC and HMBC experiments): 8.4 (C(7^1^)); 9.1 (C(18^1^)); 9.2 (C(12^1^)); 12.2 (C(2^1^)); 22.5 (C(171)); 23.8 (C(10)); 24.9 (C(81)); 30.3 (C(20)); 37.0 (C(15)); 40.3 (C(17^2^)); 52.4 (C(13^5^)); 61.9 (C(1)); 62.3 (C(6')); 67.5 (C(13^2^)); 70.3 (C(8^2^)); 71.2 (C(4')); 75.1 (C(2')); 77.6 (C(3')); 77.6 (C(5')); 103.9 (C(1')); 112.1 (C(12)); 115.2 (C(18)); 118.6 (C(3^2^)); 120.1 (C(8)); 120.3 (C(17)); 124.2 (C(16)); 124.4 (C(19)); 125.4 (C(13)); 126.9 (C(31)); 128.1 (C(3)); 128.7 (C(6)); 133.7 (C(11)); 135.4 (C(7)); 139.5 (C(9)); 156.5 (C(2)); 161.8 (C(14)); 171.3 (C(13^3^)); 174.2 (C(4)); 182.7 (C(17^3^)). ESI-MS: 845.32 (42, [M + K] + ), 829.38 (13, [M + Na] + ), 807.37 (100, *[M+*H] + ), 775.34 (15, *[M +* H - MeOH] + ), 684.24 (8, *[M +* H - ring *A] +* ), 645.29 (7, [M + H-QH_10_O_s_] + ).

*Co-injection Experiments Using Anal. HPLC.* Anal. HPLC was used to identify NCCs from freshly prepared MeOH extracts of senescent leaves of the lime tree and of maize. A yellow lime tree leaf was grounded in a mortar with 0.2 g of sea sand, frozen with liquid N_2_, and extracted with 2.5 ml of MeOH. The suspension was centrifuged at 13,000 rpm for **5** min. The clear supernatant (200 ml) was diluted with 1.3 ml of phosphate buffer (pH 7) and again centrifuged at 13,000 rpm for 4 min. Fractions of *Tc*-NCC-1 or of Zm-NCC-1 eluted (both) at a *t_R_* of *ca.* 30 min (see *Fig. 4).* For co-injection experiments, separated samples of *Tc*-NCC-1 and of Zm-NCC-1, as well as a 1:2 mixture of *Tc*-NCC-1 and Zm-NCC-1 were analyzed by anal. HPLC (see *Fig. 4).*

*Formation of*Tc*-YCC* (13) *by Oxidation of *Tc*-NCC-1 (**5**), and Data of*Tc*-YCC* (13). For further characterization of *Tc*-YCC (13) as oxidation product of *Tc*-NCC-1 (**5**), a MeOH soln. of anal. pure **5** was applied to a silica-gel TLC plate. The nearly colorless ‘spot’ acquired a brownish-red color after 10 min of irradiation with a 365-nm UV lamp (220 V, 50 Hz, SVL, VILBER LOURMAT). HPLC Analysis and a co-injection experiment with isolated *Tc*-YCC (13) showed 13 to co-elute with the yellow oxidation product of *Tc*-NCC-1 (**5**), which had an UV/VIS spectrum typical for YCCs.

*Data of 13. t_R_* 33.8 min (see main text and *Fig. 1).* UV/VIS (online, 50 mm aq. potassium phosphate (pH7)/MeOH 50:50): 247 (0.7), 313 (1.0), 413 (1.3) nm. LC/ESI-MS: 899.20 (5, [M-H + Na + K] + ), 883.20(7, [M-H + 2Na] + ), 877.27 (8, [M + K] + ), 861.27 (57, [M+Na] + ), 839.07 (32, [M+H] + ), 821.13 (100, [M+H-H_2_O] + ), 789.27 (23, [M+H-H_2_O-MeOH] + ), 659.20 (40, *[M +* H-H_2_O-MeOH]^+^), 627.27(20, [M + H- H_2_O-MeOH-C_6_H_10_O_s_] + ), 518.87 (3, [M + H- ring *A-* ring D] + ). ESI-MS (MS/ MS of the isolated protonated fragment m/z 839.07, positive ion-mode): 821.13 (100, [M+H-H_2_O] + ), 807.20 (3, [M+H-MeOH] + ), 659.13 (4, [M+H-H_2_O-C_6_H_10_O_s_] + ). ESI-MS (MS/MS of the isolated fragment at *m/z* 821.13; positive ion-mode): 789.07 (34, [M + H-H_2_O-MeOH] + ), 659.13 (100, *[M +*_5_] + ), 627.07 (10, [M+H-H_2_O-MeOH-C_6_H_10_O_s_] + ).

*Verse 902: “When from the wounded dragon / reeking flowed the blood, And therein did bathe him / the valiant knight and good, Fell down between his shoulders / full broad a linden leaf. There may he be smitten; / ‘tis cause to me of mickle grief”,* in ‘Project *Gutenberg's* The Nibelungenlied', translated into rhymed english verse in the metre of the original, translation by *George Henry Needler,* January, 2005 (e.g., EBook #7321, http://www.gutenberg.org/).
